# Supplementary material for: Saikosaponin B2, Punicalin, and Punicalagin in Vitro Block Cellular Entry of Feline Herpesvirus-1
Source: Viruses. 2024 Feb 1;16(2):231. doi: 10.3390/v16020231 (PMC10892935; doi:10.3390/v16020231)
Supplement: Supplementary file 1 [file viruses-16-00231-s001.zip › viruses-2783645-supplementary.pdf]

S1: Interpretation of nouns

$TCID_{50}$: The TCID50 represents the dilution of a virus at which 50% of a cell culture or tissue culture system becomes infected.

NMR: Nuclear Magnetic Resonance

UPLC-UV: Ultra-Performance Liquid Chromatography with Ultraviolet Detection

PFU: Plaque-Forming Unit

S2: Drugs, CAS and Purity

Selection criteria: Known to exhibit antiviral activity in vitro.

Natural small molecule drugs	CAS	Purity (%)	DOI
Isomangiferin	24699-16-9	99.82	10.1111/jfbc.13851
Diosmin	520-27-4	≥98.0	10.3390/ijms231911570
Artemitin	479-90-3	99.73	10.1002/jcb.25152
Bergenin	477-90-7	99.21	10.1021/np960074h
Coumarin	91-64-5	99.81	10.1016/j.fitote.2021.104863
Betulonic acid	4481-62-3	≥98.0	10.1016 /j.bioorg.2022.106316
Mycophenolic acid	24280-93-1	99.87	10.1159/000321483
Curcumenol	19431-84-6	99.96	10.1002/ptr.6796
Cynarin	30964-13-7	99.83	10.1016/j.antiviral.2022.105251
Umifenovir hydrochloride	131707-23-8	99.31	10.3906/sag-2106-250
Chlorogenic acid	327-97-9	99.55	10.1038/srep45723
Quercetin	117-39-5	98.03	10.3390/biom11010010
Baicalin	21967-41-9	99.17	10.1016/j.jtcme.2023.05.009
Silymarin	65666-07-1	98	10.1038/s41598-021-98949-y
Ribavirin	36791-04-5	99.8	10.1111/liv.13212
Kaempferide	491-54-3	99.35	10.1016 /j.bcp.2020.113962

Imatinib	152459-95-5	99.95	10.1016/j.cmet.2022.01.008
Isoborneol	124-76-5	≥98.0	10.1016/s0166-3542(99)00036-4
Hypericin	548-04-9	≥98.0	10.1016/j.phymed.2017.08.009
Emodin	518-82-1	99.2	10.1073/pnas.2301775120
Direct Violet 1	2586-60-9	98	10.3390/ph15091084
Punicalagin	65995-63-3	99.97	10.1002/jmv.26449
Mulberroside C	102841-43-0	99.77	10.1016/j.ejphar.2021.174204
Epigoitrin	1072-93-1	99.91	10.3389/fphar.2019.00078
Procyanidin A1	103883-03-0	99.19	10.1007/s40203-023-00143-7
Apigenin	520-36-5	99.58	10.26355/eurrev_202208_29403
LDHA-IN-4	1370290-34-8	98	10.3390/v10080407
DIM-C-pPhCO2Me	151358-48-4	99.55	10.1016/j.molcel.2021.09.016
Mitoxantrone dihydrochloride	70476-82-3	99.55	10.1186/s12866-019-1639-8
3'-Hydroxypuerarin	117060-54-5	99.95	10.1016/j.jep.2004.08.029
ICG-001	780757-88-2	99.86	10.1038/s41598-017-00282-w
Rutin	153-18-4	98.06	10.1016/j.envres.2023.115725
2-Hydroxyacetophenone	582-24-1	99.91	10.2174/011570162X261377231107110447
QS-21	141256-04-4	99.2	10.1016/0264-410x（95）98263-a
β-Cyclodextrin	7585-39-9	≥98.0	10.1002/adfm.201908788
Clovamide	53755-02-5	98.48	10.3389/fpls.2020.617520
Azadirachtin B	106500-25-8	99.12	10.5650/jos.58.581
Etoposide	33419-42-0	99.94	10.1111/bjh.15988
Silibinin	802918-57-6	99.91	10.1016/j.bbrep.2018.03.003
Ivermectin	70288-86-7	98.01	10.1056/NEJMoa2201662

Hesperidin	520-26-3	99.19	10.3851/IMP3235
Kanamycin (sulfate)	25389-94-0	≥98.0	10.1016/0166-3542（91）90064-x
Rottlerin	30169	98.15	10.1038/s41564-021-00968-y
Tauroursodeoxycholate	146052-22-2	99.13	10.1186/s12964-023-01081-9
Tunicamycin	11089-65-9	99.96	10.1016/j.micpath.2020.104586
Amphotericin B	1397-89-3	≥98.0	10.1016 /j.celrep.2013.10.033
Oxytetracycline	79-57-2	99.05	10.1073/pnas.2024420118
D-Sorbitol	50-70-4	≥98.0	10.1016/j.phyplu.2022.100402
Lanatoside C	17575-22-3	99.81	10.1016/j.phymed.2023.155308
Escin	6805-41-0	98	10.1038/s41598-023-36871-1
N-Acetylneuraminic acid	131-48-6	≥98.0	10.1016/j.virusres.2016.05.031
(-)-Epicatechin gallate	1257-08-5	98.39	10.2174/0929867324666161123091010
Geniposide	24512-63-8	99.89	10.1016/j.jep.2023.116745
Bergenin	477-90-7	99.21	10.1016/j.jep.2005.01.056
Isochlorogenic acid A	2450-53-5	99.54	10.1248/cpb.48.1796
3,4-Dicaffeoylquinic acid	14534-61-3	98.64	10.1016/j.fitote.2015.03.024
4,5-Dicaffeoylquinic acid	57378-72-0	99.98	10.1073/pnas.93.13.6326
Dihydromyricetin	27200-12-0	98.1	10.1186/s13567-023-01184-8
Quercetin (dihydrate)	6151-25-3	≥96.0	10.2147/DDDT.S123340
Glycitin	40246-10-4	99.84	10.1016/j.intimp.2019.105749
Glycyrrhizic acid	1405-86-3	≥98.0	10.1016/j.bmcl.2019.126645
Dipotassium glycyrrhizinate	68797-35-3	99.51	10.2174/1389557519666190119111125
Theaflavin	1011850	99.06	10.3389/fphar.2020.514313
Saikosaponin B2	58316-41-9	98.94	10.1016/j.jhep.2014.10.040

Sennoside A	81-27-6	99.71	10.1016/j.phymed.2016.08.001
D-Mannitol	69-65-8	≥98.0	10.3390/ph11030073
Hyperoside	482-36-0	99.56	10.3389/fphar.2019.01272
L-Chicoric Acid	70831-56-0	99.6	10.1016/j.virol.2004.06.005
Corilagin	23094-69-1	99.87	10.1515/tnsci-2018-0003
Dryocrassin ABBA	12777-70-7	98	10.3389/fmicb.2015.00592
Deapioplatycodin D	78763-58-3	≥99.0	10.1155/2013/560417
Maslinic acid	4373-41-5	≥98.0	10.3390/ijms242316617
Punicalin	65995-64-4	98.84	10.1016/j.antiviral.2016.08.026
Scutellarin	27740-01-8	98.56	10.1016/j.bbrc.2005.06.166
Scutellarein	529-53-3	99.25	10.1016 /j.cbi.2020.109211
Catalpol	2415-24-9	99.98	10.1016/j.cbi.2021.109625
Catechin	152-23-4	99.57	10.1016/j.phyplu.2022.100402
Isoquercetin	482-35-9	99.87	10.3389/fphar.2022.830205
Theaflavin 3,3'-digallate	30462-35-2	99.73	10.3389/fphar.2020.514313
Chebulagic acid	23094-71-5	99.67	10.3389/fmicb.2020.00182
Maltose	69-79-4	≥98.0	10.1128/JVI.68.9.6029-6037.1994
Swertianolin	23445-00-3	99.54	10.3389/fpls.2022.809497
Polygalasaponin XXXI	79103-90-5	96.19	10.1016/s0264-410x（01）00215-8
Trilobatin	4192-90-9	99	10.1002/1873-3468.13113
Cichoriin	531-58-8	99.96	10.3390/biom11020216
Betulinic acid	472-15-1	≥98.0	10.1016/s0968-0896（97）00158-2
5-Aminosalicylic Acid	89-57-6	≥98.0	10.1136/gutjnl-2021-324397
Berberine (chloride)	633-65-8	99.66	10.3390/v13122437

Triptolide	38748-32-2	99.86	10.1002/iid3.667
α-Lipoic Acid	1077-28-7	99.86	10.1007/BF01649442
Methyl gallate	99-24-1	99.96	10.1007/BF01128976
Dehydroabietic acid	1740-19-8	99.75	10.1016 /j.bmc.2003.10.013
Limonin	1180-71-8	98.68	10.1055/s-2003-45099
PCL 016	98-98-6	99.02	10.1016/j.xcrm.2023.101127
2,2'-Anhydrouridine	3736-77-4	≥98.0	10.1016/0006-2952（87）90570-3
Acridone	578-95-0	≥98.0	10.1186/s12929-015-0134-2
Clofoctol	37693-01-9	99.75	10.1371/journal.ppat.1010498
Spermine	71-44-3	98.36	10.1016/j.immuni.2023.01.001
Euparin	532-48-9	≥99.0	10.1155/2013/402364
Gymnemagenin	22467-07-8	99.44	10.1007/s12010-014-0754-0
3-Methoxyflavone	7245-02-5	99.88	10.1128 /JVI.61.10.3319-3321.1987
Hederasaponin B	36284-77-2	98.51	10.4062/biomolther.2013.108
Verbenalin	548-37-8	99.91	10.1007/s10668-021-01373-5
Cephalotaxine	24316-19-6	99.52	10.1016/j.bbrc.2019.12.012
Xanthone	90-47-1	99.97	10.1155/2020/3972390
Arctigenin	7770-78-7	99.69	10.1248/bpb.33.1199
DL-Serine	302-84-1	≥98.0	10.1111/j.1439-0434.1967.tb02294.x
Ginsenoside Rb2	11021-13-9	98.26	10.3389/fvets.2021.764909
Daphnoretin	2034-69-7	99.83	10.1016/0006-2952（96）00420-0
Yadanziolide A	95258-14-3	99.41	10.1021/jf903434h
Alisol F	155521-45-2	≥99.0	10.1055/s-2006-947178
Isoxanthohumol	521-48-2	99.9	10.1016 /j.phymed.2023.155176

Camphor	76-22-2	≥98.0	10.1002/ardp.202100038
Alopearine	56293-29-9	≥98.0	10.1016/s0378-8741（01）00389-0
Cytarabine	147-94-4	99.96	10.1128/AAC.4.4.439
Lapachol	84-79-7	99.06	10.1016/s0968-0896（02）00542-4
Octyl gallate	1034-01-1	98.01	10.1016/j.vetmic.2023.109743
D-Pinitol	10284-63-6	≥98.0	10.1002/cbdv.200690114
Amentoflavone	1617-53-4	99.81	10.1111/1348-0421.13064
Curcumol	4871-97-0	99.5	10.1016/j.bcp.2021.114742
Angelicin	523-50-2	99.86	10.1016/j.antiviral.2013.07.009
Xanthohumol	6754-58-1	99.97	10.1055/s-0033-1360172
Sophocarpine	6483-15-4	98.63	10.3892/etm.2017.4958
Andrographolide	5508-58-7	99.89	10.1016 /j.antiviral.2016.12.014
4-Hydroxycoumarin	1076-38-6	99.96	10.3390/molecules16076023
alpha-Mangostin	6147-11-1	99.64	10.3390/molecules27217362
Picroside II	39012-20-9	99.61	10.1021/np900549e
Gramine	87-52-5	99.63	10.3390/v13081433
Oxyresveratrol	29700-22-9	98.87	10.1208/s12249-012-9828-x
Brefeldin A	20350-15-6	99.87	10.1128/jvi.77.5.3204-3216.2003
Aloe emodin	481-72-1	98.32	10.1016/j.ijantimicag.2008.04.018
Glyceryl monocaprate	26402-22-2	≥98.0	10.3390/pathogens12101193
Licoflavone B	91433-17-9	99.91	10.1080 /07391102.2021.1959401
Pristinamycin IA	3131-03-1	98.91	10.1016/0922-4106（95）90193-0
Digitoxin	71-63-6	99.36	10.2147/JEP.S273120
Euscaphic acid	53155-25-2	98.34	10.1016/j.jep.2017.08.005

Isosteviol	27975-19-5	≥98.0	10.1016/j.phytochem.2013.12.014
Neobavaisoflavone	41060-15-5	99.91	10.1016 /j.bbrc.2020.11.083
Allicin	539-86-6	97.74	10.3390/nu12030872
Berberine sulfate	633-66-9	≥98.0	10.1016/j.biochi.2019.04.008
SIBA	35899-54-8	99.66	10.1016/j.antiviral.2013.06.001
FIT-039	1113044-49-7	99.56	10.1172/JCI73805
Isoborneol	124-76-5	≥98.0	10.1016/s0166-3542（99）00036-4
Zerumbone	471-05-6	99.87	10.1016/j.heliyon.2021.e06710
(2S)-Isoxanthohumol	70872-29-6	99.77	10.1016/s0166-3542（03）00155-4
BIO-acetoxime	667463-85-6	≥98.0	10.1007/s00705-013-1629-3
ML324	1222800-79-4	98.54	10.3390/v15010163
Guanosine	118-00-3	99.31	10.1016 /j.phymed.2023.155020
OG-L002	1357302-64-7	99.81	10.3389/fvets.2018.00034
Verbascoside	61276-17-3	99.83	10.1039/c6fo00335d
Salubrinal	405060-95-9	99.69	10.1016/j.virol.2008.06.028
Ginsenoside Rb1	41753-43-9	98.75	10.1016 /j.jep.2020.113401
PIK-93	593960-11-3	99.81	10.1128/JVI.02249-10
Trifluridine	70-00-8	99.98	10.2165/00003495-198223050-00001
(Z)-Capsaicin	25775-90-0	99.68	10.1038/s41401-023-01111-9
Floxuridine	50-91-9	99.95	10.1089/aid.1996.12.965
1-Docosanol	661-19-8	≥98.0	10.1067/mjd.2001.116215
Idoxuridine	54-42-2	99.70	10.2460/ajvr.2004.65.399
Brivudine	69304-47-8	99.4	10.1358/dot.2003.39.5.740221
Valpromide	2430-27-5	≥98.0	10.1016/j.antiviral.2019.05.006

Oxytetracycline hydrochloride	2058-46-0	98.73	10.1073/pnas.2024420118
S-Methylisothiourea sulfate	867-44-7	≥98.0	10.1002/eji.200838885
5'-AMP monohydrate	18422-05-4	99.07	10.1016/0166-3542（83）90023-2

S3: qPCR primers sequences, probe sequences, amplification procedures, and FHV-1 amplification standard curves

Forward primer: 5`-GCAGATCTACACATCAGA-3`

Reversed primer: 5`-GGGTGATGTTAAATGTGG-3`

Probe:5`-FAM-CTCGCCTGAGATGACGGTCC-BHQ1-3`

Amplification procedures： 95 °C 30 S; 95 °C 5 S ,60 °C 30 S, Fluorescence signals were collected in the second step of each cycle (60 °C 30 S) for a total of 40 cycles.

Standard curves: $y(Cq) = -3.1702 * X(Log\ Quantity) + 39.72$ $R^2 > 0.999$

S4: The cytoxicity data for the compounds

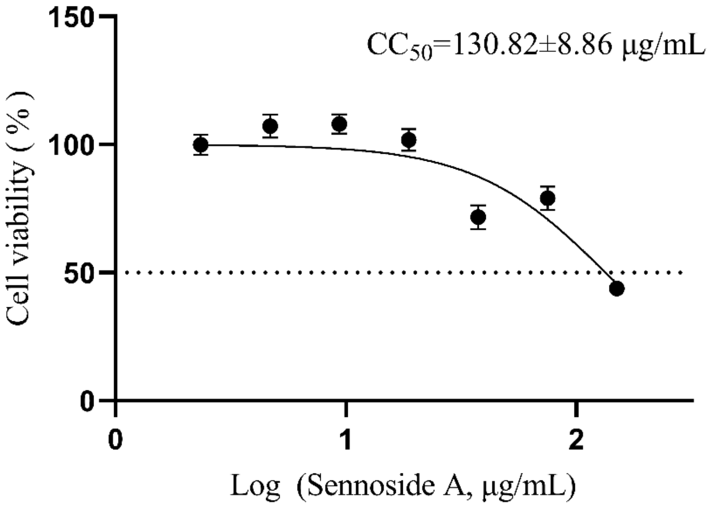

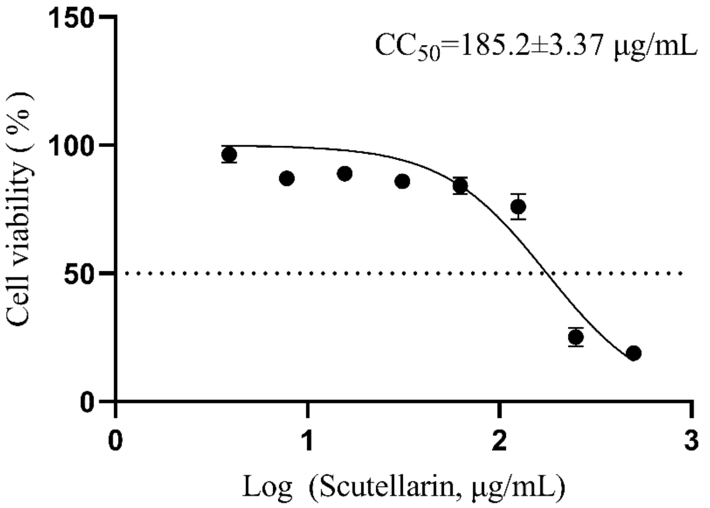

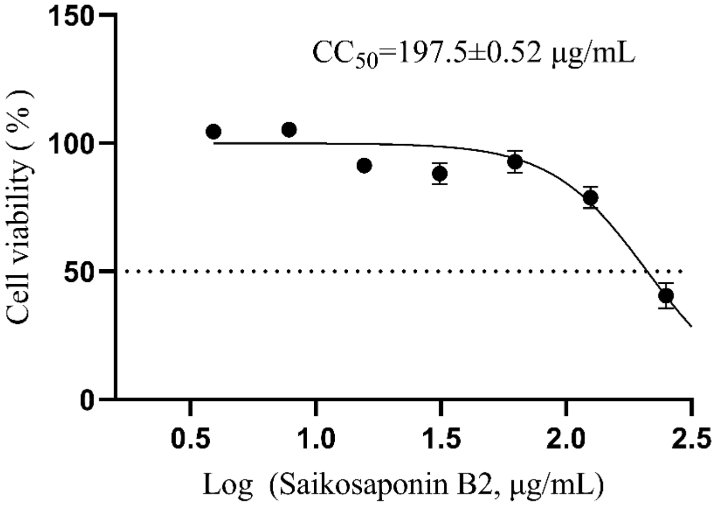

CC50=197.5±0.52 μg/mL
Cell viability (%)
150
100
50
0
0.5
1.0
1.5
2.0
2.5
Log (Saikosaponin B2, μg/mL)

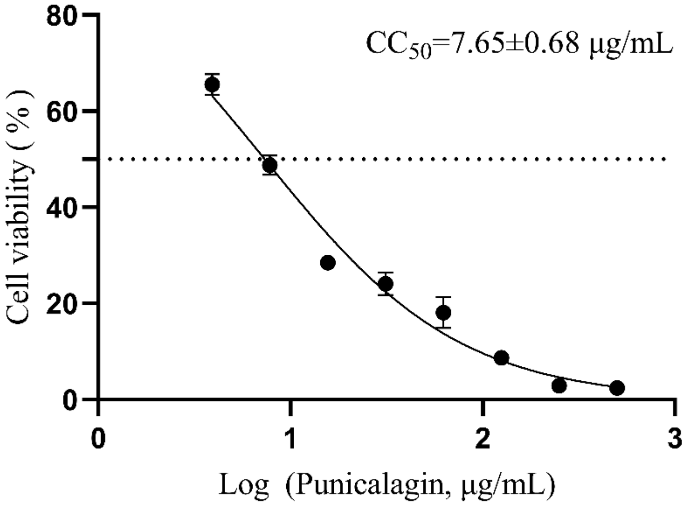

CC50=7.65±0.68 μg/mL
Cell viability (%)
80
60
40
20
0
0
1
2
3
Log (Punicalagin, μg/mL)

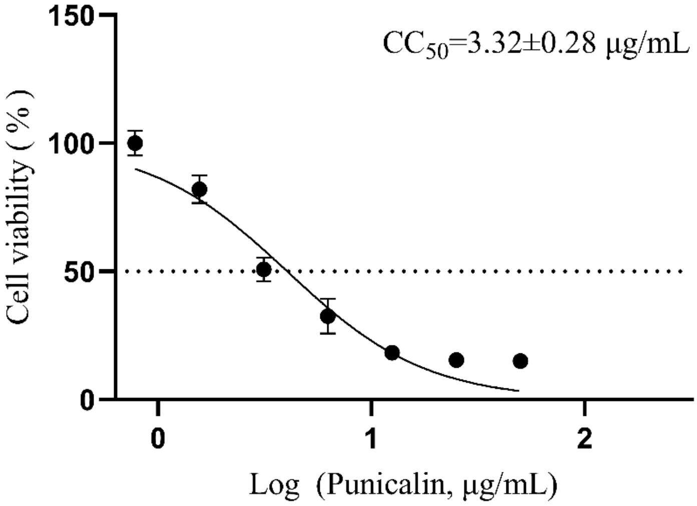

CC50=3.32±0.28 μg/mL
Cell viability (%)
150
100
50
0
0
1
2
Log (Punicalin, μg/mL)

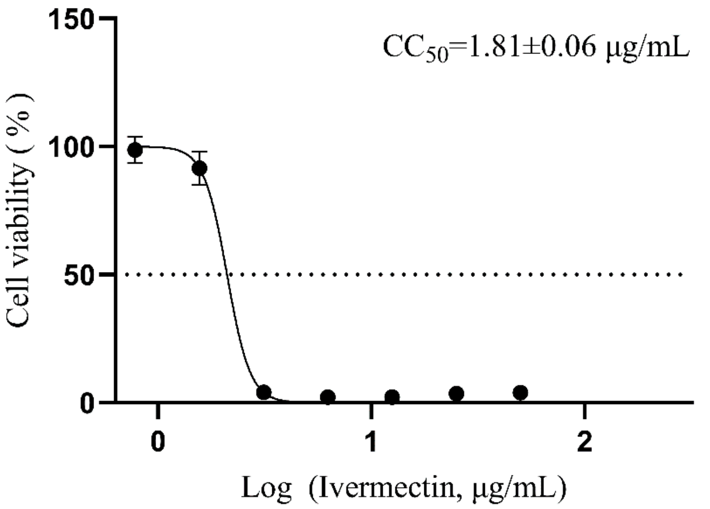

CC50=1.81±0.06 μg/mL
Cell viability (%)
150
100
50
0
0
1
2
Log (Ivermectin, μg/mL)

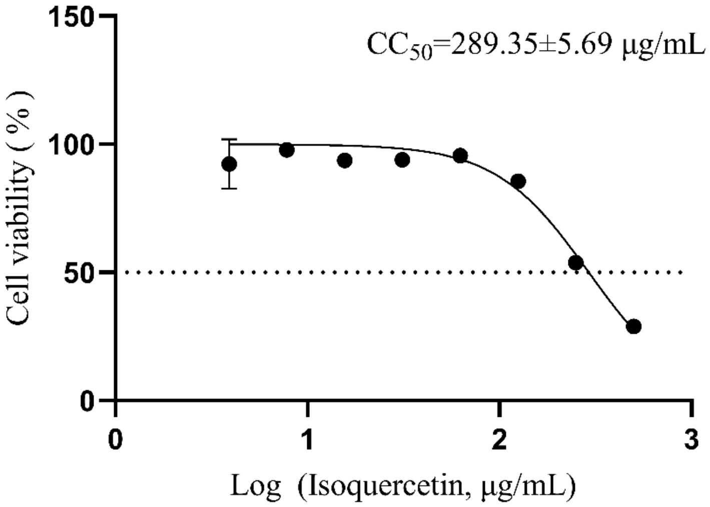

CC50=289.35±5.69 μg/mL
Cell viability (%)
150
100
50
0
0
1
2
3
Log (Isoquercetin, μg/mL)

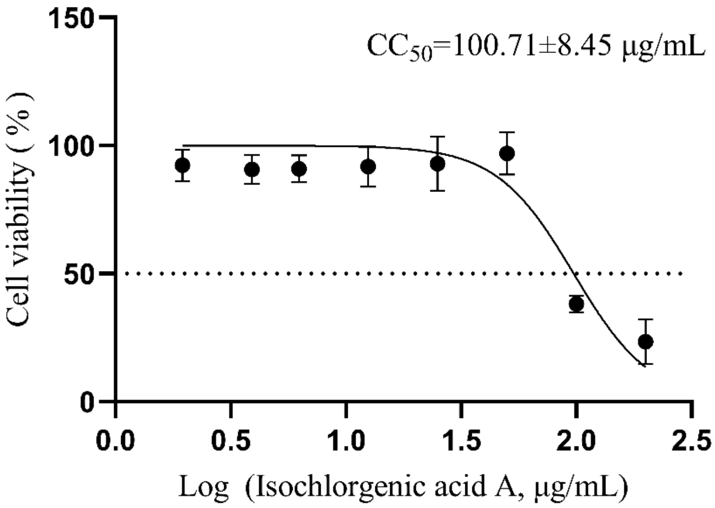

CC50=100.71±8.45 μg/mL
Cell viability (%)
150
100
50
0
0.0
0.5
1.0
1.5
2.0
2.5
Log (Isochlorgenic acid A, μg/mL)

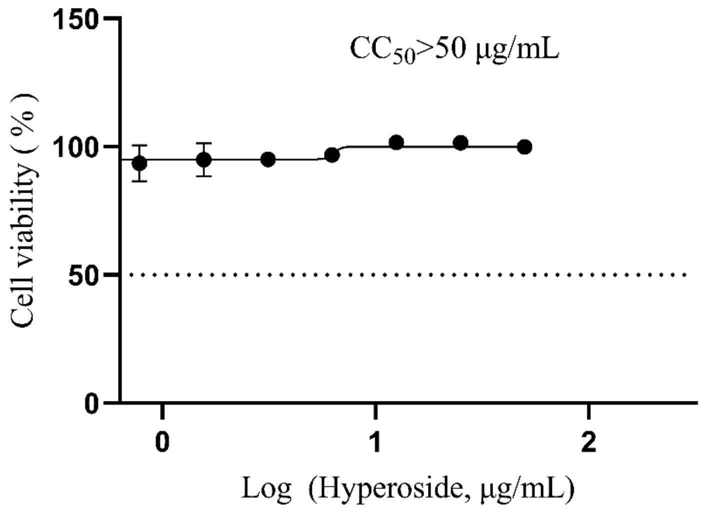

CC_{50}>50 μg/mL
Cell viability (%)
150
100
50
0
0
1
2
Log (Hyperoside, μg/mL)

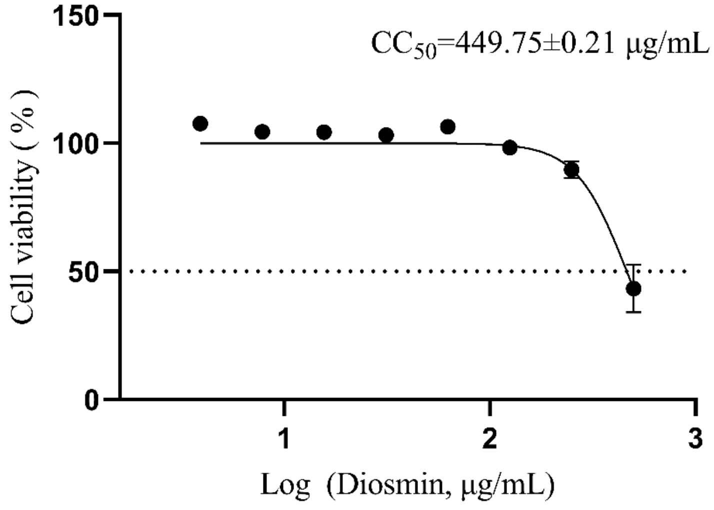

CC_{50}=449.75±0.21 μg/mL
Cell viability (%)
150
100
50
0
1
2
3
Log (Diosmin, μg/mL)

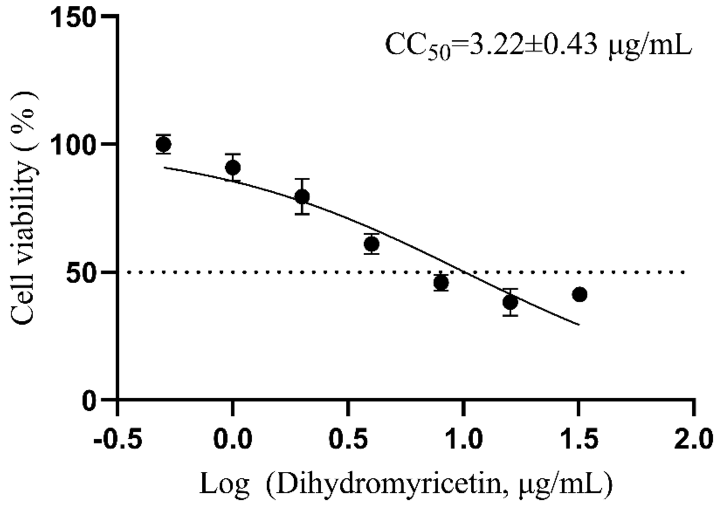

CC_{50}=3.22±0.43 μg/mL
Cell viability (%)
150
100
50
0
-0.5
0.0
0.5
1.0
1.5
2.0
Log (Dihydromyricetin, μg/mL)

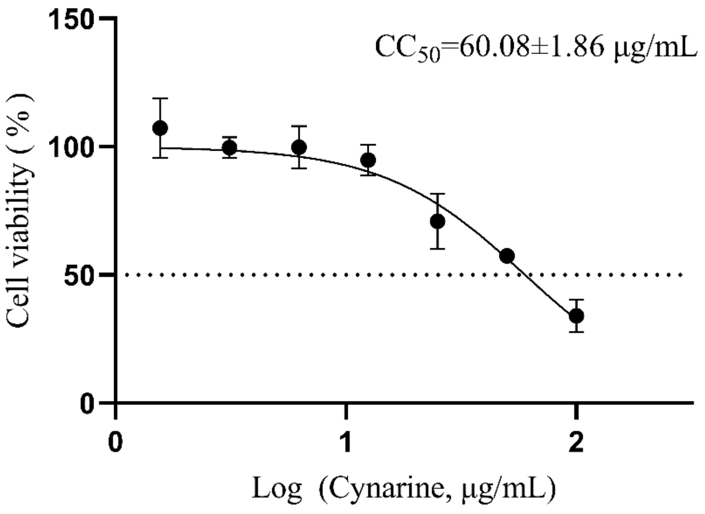

CC50=60.08±1.86 μg/mL
Cell viability (%)
150
100
50
0
0
1
2
Log (Cynarine, μg/mL)

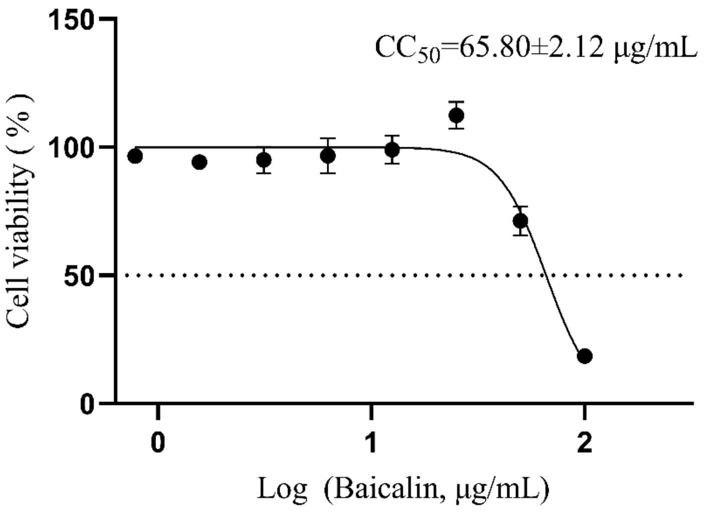

CC50=65.80±2.12 μg/mL
Cell viability (%)
150
100
50
0
0
1
2
Log (Baicalin, μg/mL)

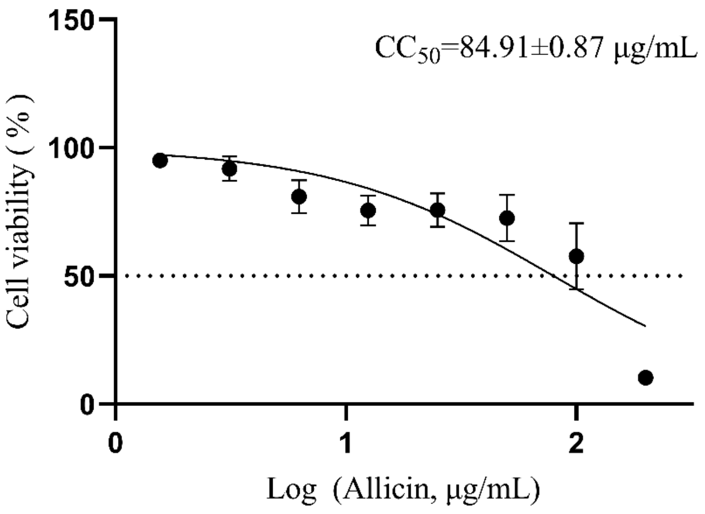

CC50=84.91±0.87 μg/mL
Cell viability (%)
150
100
50
0
0
1
2
Log (Allicin, μg/mL)

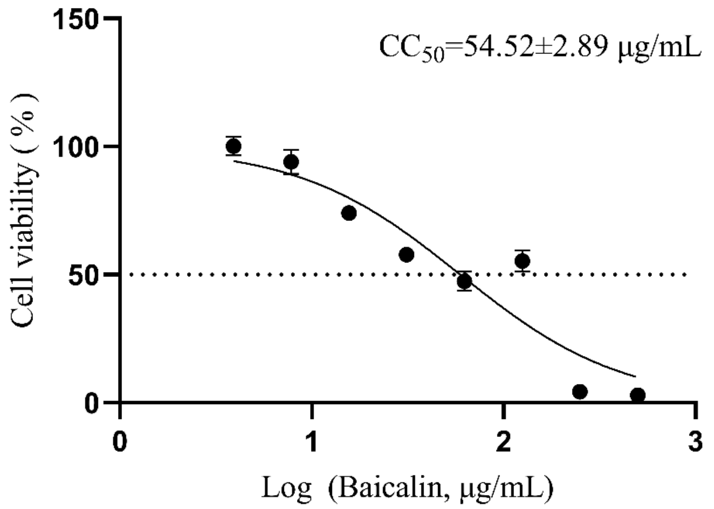
150
100
50
0
Cell viability (%)
CC$_{50}$=54.52±2.89 μg/mL
0
1
2
3
Log (Baicalin, μg/mL)

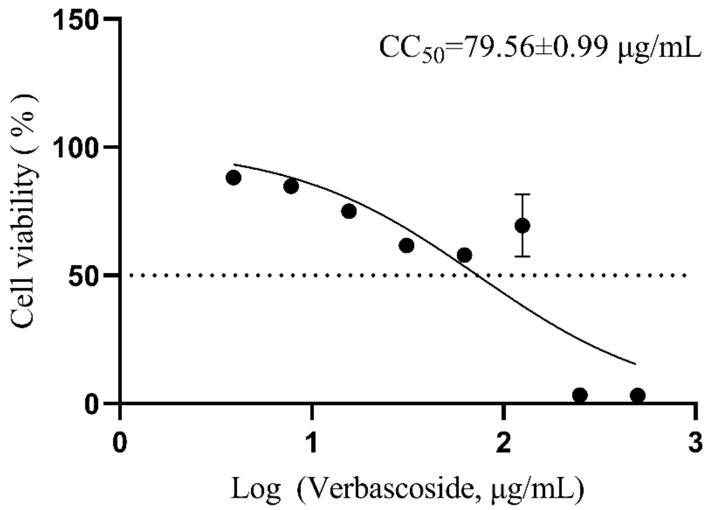
150
100
50
0
Cell viability (%)
CC$_{50}$=79.56±0.99 μg/mL
0
1
2
3
Log (Verbascoside, μg/mL)
